# The Prevalence of Abnormal Uterine Bleeding and Its Subtypes Among Adolescent Girls in India: A Systematic Review and Meta-Analysis

**DOI:** 10.7759/cureus.112607

**Published:** 2026-07-13

**Authors:** Ekta Krishna, Madhusudan P Singh, Naveen Suthar, Shamshad Ahmad, Haripriya H, Pragya Kumar, Purnima Singh, Vijay Kumar, Sanjay Pandey

**Affiliations:** 1 Community and Family Medicine, ESIC Medical College and Hospital, Bihta, Patna, IND; 2 Pharmacology and Therapeutics, All India Institute of Medical Sciences, Deoghar, Deoghar, IND; 3 Community and Family Medicine, All India Institute of Medical Sciences, Patna, Patna, IND; 4 Obstetrics and Gynaecology, Netaji Subhas Medical College and Hospital, Adityapur, Jamshedpur, IND; 5 Radiodiagnosis, Indira Gandhi Institute of Medical Sciences, Patna, IND

**Keywords:** abnormal uterine bleeding (aub), adolescents' health, female reproductive health, from india, menorrhagia, menstrual disorders, metrorrhagia, oligomenorrhea, primary dysmenorrhea, systematic review and meta analysis

## Abstract

This study aimed to determine the national pooled prevalence of abnormal uterine bleeding (AUB), its subtypes, and dysmenorrhea among adolescent girls in India. We conducted a systematic review and meta-analysis following the PRISMA (Preferred Reporting Items for Systematic Reviews and Meta-Analyses) guidelines, searching PubMed, Embase, and Web of Science from inception to December 4, 2024. Twenty nine observational studies involving adolescent girls aged 10-19 years, with sample sizes ranging from 127 to 2,000, conducted across India, were included. Due to high heterogeneity, pooled prevalence estimates were calculated using random-effects models. The pooled prevalence of AUB was 18.0% (95% confidence interval (CI), 14.0%-22.0%). Among AUB subtypes, oligomenorrhea (24%, 95% CI: 15%-34%) and metrorrhagia (24%, 95% CI: 4%-44%) were the most common, followed by menorrhagia (18%, 95% CI: 14%-23%). Additionally, the pooled prevalence of dysmenorrhea was notably high at 53% (95% CI: 46%-61%, I² = 99.4%). Significant heterogeneity (I² > 99.1%) and regional disparities in prevalence were observed across all analyses. This study found that about one in five adolescent girls in India experience AUB. These findings support the need for future studies to evaluate the effectiveness, accuracy, and feasibility of screening strategies for their widespread implementation.

## Introduction and background

Abnormal uterine bleeding (AUB), according to the FIGO (International Federation of Gynaecology and Obstetrics) System 1, is defined as bleeding from the uterine corpus that is abnormal in frequency, regularity, duration, or volume in the absence of pregnancy [1,2]. Rather than representing a single disease entity, AUB encompasses a spectrum of abnormal bleeding patterns including oligomenorrhea, polymenorrhea, hypomenorrhea, hypermenorrhea, metrorrhagia, and menorrhagia [2,3]. The latest classification system, the FIGO PALM-COEIN system, classifies AUB based on its underlying etiology into structural causes (Polyp, Adenomyosis, Leiomyoma, Malignancy, and Hyperplasia) and non-structural causes (Coagulopathy, Ovulatory dysfunction, Endometrial, Iatrogenic, and Not otherwise classified) [2]. AUB is one of the most prevalent gynecological conditions among adolescent girls and has a significant impact on their physical health, mental health, and academic performance.

Persistent AUB can lead to complications such as anemia, depression, and long-term reproductive problems. It may also mask other health problems, such as polycystic ovarian syndrome (PCOS), thyroid disorders, and bleeding disorders such as von Willebrand disease [4]. In teenagers, the hypothalamic-pituitary-ovarian (HPO) axis is frequently underdeveloped, resulting in anovulatory cycles and irregular bleeding patterns during the early post-menarchal years [5]. Although transient anomalies are common, persistent abnormalities lasting beyond two to three years after menarche require clinical assessment [6]. Despite this, diagnostic delays are common in India because of cultural stigmas, limited awareness among women, and inadequate healthcare systems [7]. For example, up to 90% of women with menorrhagia may have underlying bleeding disorders [8], yet screening rates remain extremely low [9].

Likewise, oligomenorrhea and other subtypes of AUB are significant manifestations associated with PCOS. One study has linked this symptom to an elevated risk of cardiovascular disease (CVD), coronary heart disease (CHD), and myocardial infarction (MI) in women [10]. These menstrual disorders often go unrecognized, yet they may have significant long-term consequences for the reproductive health of adolescent girls. Dysmenorrhea is another common menstrual disorder affecting adolescents [11]. Although dysmenorrhea is not classified as a clinical bleeding pattern of AUB according to the FIGO classification system, it frequently coexists with AUB, thereby increasing the overall burden of menstrual morbidity. Despite their high prevalence, both AUB and dysmenorrhea remain underreported and inadequately managed in many settings.

Improving awareness of menstrual health problems among adolescents in India requires robust estimates of the prevalence of AUB and its clinical subtypes. Accurate assessment of the burden of these conditions is essential for evidence-based healthcare planning, resource allocation, and the development of targeted public health interventions. Although numerous studies have investigated AUB and its clinical subtypes among Indian adolescents, their findings vary considerably because of differences in study methodologies, diagnostic criteria, and geographic representation. Therefore, we conducted a comprehensive systematic review and meta-analysis to estimate the pooled prevalence of AUB and its clinical subtypes among adolescent girls in India. In addition, we conducted a secondary analysis to estimate the pooled prevalence of dysmenorrhea to provide a broader overview of menstrual health problems in this population.

This study has been published as a preprint on www.medrxiv.org with DOI: https://doi.org/10.1101/2025.10.24.25338610; website link: https://www.medrxiv.org/content/10.1101/2025.10.24.25338610v1 on October 25, 2025.

## Review

Methods

Design

The goal of this systematic review was to determine the overall rate of AUB and its subtypes among teenage girls in India, in accordance with the 2020 Preferred Reporting Items for Systematic Reviews and Meta-Analyses (PRISMA) guidelines. The protocol for this review was also registered with the International Prospective Register of Systematic Reviews (PROSPERO; registration number: CRD42024573458).

Data Sources and Search Strategy

We developed a thorough search strategy to identify all peer-reviewed studies for our systematic review and meta-analysis across three electronic databases: PubMed, Embase, and Web of Science. We included studies published in English from database inception through December 4, 2024. This review considered only the symptom-based definition of AUB subtypes as defined by the FIGO System 1. Most epidemiological studies conducted in India are community-based studies that do not use the PALM-COEIN system due to its reliance on clinical investigations such as imaging and biopsy, which are not feasible in school or community settings.

A systematic search of the literature was conducted using a combination of controlled vocabulary (MeSH/Emtree terms) and keywords associated with four principal concepts: (1) menstrual disorders (e.g., oligomenorrhea, polymenorrhea, menorrhagia, metrorrhagia, etc.), (2) adolescent populations (e.g., "adolescent," "school-going girls," etc.), (3) the geographical focus (India), and (4) epidemiological terms (e.g., prevalence, burden) (Table 1). The search identified 514 records from PubMed, 326 from Embase, and 183 from Web of Science. All searches included studies published from database inception through December 4, 2024, across the three databases, and all identified records were included for abstract screening. Before the screening process began, the retrieved records were imported into Rayyan software to remove duplicate records.

**Table 1:** Search strategy for the review

Database	No.	Search query	Results
PubMed			
	#1	(((((((((((((((((((((((Premenstrual syndrome[MeSH Terms]) OR (Premenstrual syndrome)) OR ((inter-menstrual bleeding[MeSH Terms]) OR (inter-menstrual bleeding))) OR ((Irregular menses) OR (Irregular menses[MeSH Terms]))) OR ((abundant blood loss[MeSH Terms]) OR (abundant blood loss.))) OR (menstrual discomfort)) OR (menstrual discomfort)) OR (((Irregular menses[MeSH Terms])) OR (Irregular menses))) OR ((menstrual morbidities) OR (menstrual morbidities[MeSH Terms]))) OR ((menstrual irregularities[MeSH Terms]) OR (menstrual irregularities))) OR (menstrual disturbances)) OR ((Menstrual abnormalities[MeSH Terms]) OR (Menstrual abnormalities))) OR ((Hypermenorrhea) OR (Hypermenorrhea[MeSH Terms]))) OR ((Oligomenorrhea[MeSH Terms]) OR (Oligomenorrhea))) OR ((Metrorrhagia) OR (Metrorrhagia[MeSH Terms]))) OR ((menstruation-related disorder) OR (menstruation-related disorder[MeSH Terms]))) OR ((abnormal uterine bleeding[MeSH Terms]) OR (abnormal uterine bleeding))) OR ((Irregular menstrual cycle) OR (Irregular menstrual cycle[MeSH Terms]))) OR (((Hypomenorrhea)) OR (Hypomenorrhea[MeSH Terms]))) OR ((Polymenorrhea[MeSH Terms]) OR (Polymenorrhea))) OR ((Menorrhagia) OR (Menorrhagia[MeSH Terms]))) OR ((Dysmenorrhea[MeSH Terms]) OR (Dysmenorrhea))) OR ((Polycystic Ovarian Syndrome) OR (Polycystic Ovarian Syndrome[MeSH Terms]))) AND ("india"[All Fields])	83,332
	#2	"adolescent"[MeSH Terms] OR "adolescent"[All Fields] OR "school going girls"[All Fields] OR "teenage"[All Fields] OR "young women"[All Fields] OR "indian adolescent girls"[All Fields] OR "high school girls"[All Fields] OR "college going students"[All Fields] OR "college going women"[All Fields]	2,399,956
	#3	"india"[All Fields]	867,878
	#4	((prevalence) OR prevalence[MeSH Terms]) OR burden	3,961,025
	#4	#1 AND #2 AND #3 AND #4	514
Web of Science			
	#1	TS=(PCOS OR Dysmenorrhea OR Menorrhagia OR Polymenorrhea OR Hypomenorrhea OR "Irregular menstrual cycle" OR "abnormal uterine bleeding" OR "menstruation-related disorder" OR Metrorrhagia OR Oligomenorrhea OR Hypermenorrhea OR "Menstrual abnormalities" OR "menstrual disturbances" OR "menstrual irregularities" OR "menstrual morbidities" OR Polymenorrhagia OR "menstrual discomfort" OR "abundant blood loss" OR "Dysfunctional uterine bleeding" OR endometrosis OR "Irregular menses" OR "inter-menstrual bleeding" OR "Menstrual Pain" OR "Heavy bleeding" OR "Premenstrual syndrome")	85,505
	#2	TS=("school going girls" OR "Undergraduate student" OR "young women" OR "Indian adolescent girls" OR "high school girls" OR "college going students" OR "adolescent")	3,461,887
	#3	TS=(India OR Indian)	756,314
	#4	TS=(prevalence OR burden)	2,377,045
	#5	#1 AND #2 AND #3 AND #4	183
EMBASE			
	#1	pcos OR ('dysmenorrhea'/exp OR 'dysmenorrhea') OR ('menorrhagia'/exp OR 'menorrhagia') OR ('polymenorrhea'/exp OR 'polymenorrhea') OR ('oligomenorrhea'/exp OR 'oligomenorrhea') OR ('abnormal uterine bleeding'/exp OR 'abnormal uterine bleeding') OR 'menstruation-related disorder' OR ('metrorrhagia'/exp OR 'metrorrhagia') OR ('oligomenorrhea'/exp OR 'oligomenorrhea') OR ('hypermenorrhea'/exp OR hypermenorrhea) OR 'menstrual abnormalities' OR 'menstrual disturbances' OR 'menstrual irregularities' OR 'menstrual morbidities' OR ('polymenorrhagia'/exp OR 'polymenorrhagia') OR 'irregular menses' OR 'menstrual discomfort' OR 'abundant blood loss' OR 'polycystic ovarian syndrome' OR 'dysfunctional uterine bleeding' OR endometrosis OR 'inter-menstrual bleeding' OR 'menstrual pain' OR 'heavy bleeding' OR ('premenstrual syndrome'/exp OR 'premenstrual syndrome')	84,240
	#2	'adolescent' OR ('school child'/exp OR 'school child') OR 'school going girls' OR ('undergraduate student'/exp OR 'undergraduate student') OR 'young women' OR 'indian adolescent girls' OR 'high school girls' OR 'college going students'	2,453,036
	#3	('india'/exp OR 'india') OR ('indian'/exp OR 'indian')	1,645,235
	#4	('prevalence'/exp OR 'prevalence') OR ('burden'/exp OR 'burden')	2,005,876
	#5	#1 AND #2 AND #3 AND #4	326

*Eligibility Criteria* 

We selected studies based on predefined eligibility criteria. We included observational studies (cross-sectional, cohort, and case-control) and randomized controlled trials that reported baseline prevalence data on outcomes among adolescent girls aged 10 to 19 years in India. For this systematic review, an AUB case was defined according to the FIGO System 1 classification, which describes abnormal uterine bleeding based on the clinical pattern of menstrual bleeding (frequency, regularity, duration, and volume). Therefore, studies reporting one or more clinical bleeding pattern abnormalities, including oligomenorrhea, polymenorrhea, hypomenorrhea, hypermenorrhea, menorrhagia, or metrorrhagia, were considered to report AUB cases (Table 2). 

**Table 2:** Operational definitions used for data extraction Operational definitions for the AUB clinical subtypes included in this review were based on the definitions provided in DC Dutta's Textbook of Gynaecology AUB: abnormal uterine bleeding

Sr. No.	AUB clinical subtype	Operational definition
1.	Oligomenorrhea	Infrequent and irregular menstrual periods, with menstrual cycles lasting more than 35 days
2.	Polymenorrhea	Menstrual cycles occur more frequently than normal, with cycle intervals of less than 21 days
3.	Hypermenorrhea	Prolonged menstrual bleeding occurring at regular intervals, with bleeding lasting more than 8 days
4.	Hypomenorrhea	Reduced menstrual blood flow or shortened duration of menstruation, with bleeding lasting less than 2 days
5.	Menorrhagia	Excessive uterine bleeding occurs at regular intervals, with more than 80 ml of blood loss per menstrual cycle
6.	Metrorrhagia	Uterine bleeding occurs at irregular intervals, particularly between expected menstrual periods

We did not classify cases according to the FIGO PALM-COEIN (System 2) classification, as that framework categorizes the underlying structural and non-structural etiologies of AUB rather than the clinical bleeding pattern. We did not include studies that focused on pregnant adolescents. This review was restricted to studies conducted among Indian populations and excluded studies that did not have extractable prevalence data relevant to our study outcomes. We only considered publications available in English. Additionally, case reports, review articles, and non-peer-reviewed publications were excluded. We only included papers that were published from database inception through December 4, 2024.

Data Extraction Using Rayyan Software

Two independent reviewers screened the publications using Rayyan software. The reviewers systematically evaluated the titles and abstracts against the eligibility criteria to assess study eligibility and methodological rigor. Discrepancies in article selection and data extraction were addressed through consultation with a third reviewer. We then obtained the full texts of potentially eligible papers and uploaded them to Rayyan software. The retrieved articles were meticulously re-evaluated to ascertain their eligibility, and pertinent data were meticulously collected.

We utilized a standardized data extraction form to gather information about the study's characteristics (author, year, state, study design, sample size, and study setting), population (sample characteristics, including sample size and age distribution), and prevalence estimates for each type of AUB (operational definitions used for each type, etc.). Rayyan's capabilities, such as blind review mode, tagging, and collaborative tools, facilitated the efficient screening of large sets of records. All excluded studies were documented with explanations for their exclusion to maintain transparency. The software's export capability made it possible to generate PRISMA-standard flowcharts to illustrate the research selection process.

Data Analysis

**Figure 1:**
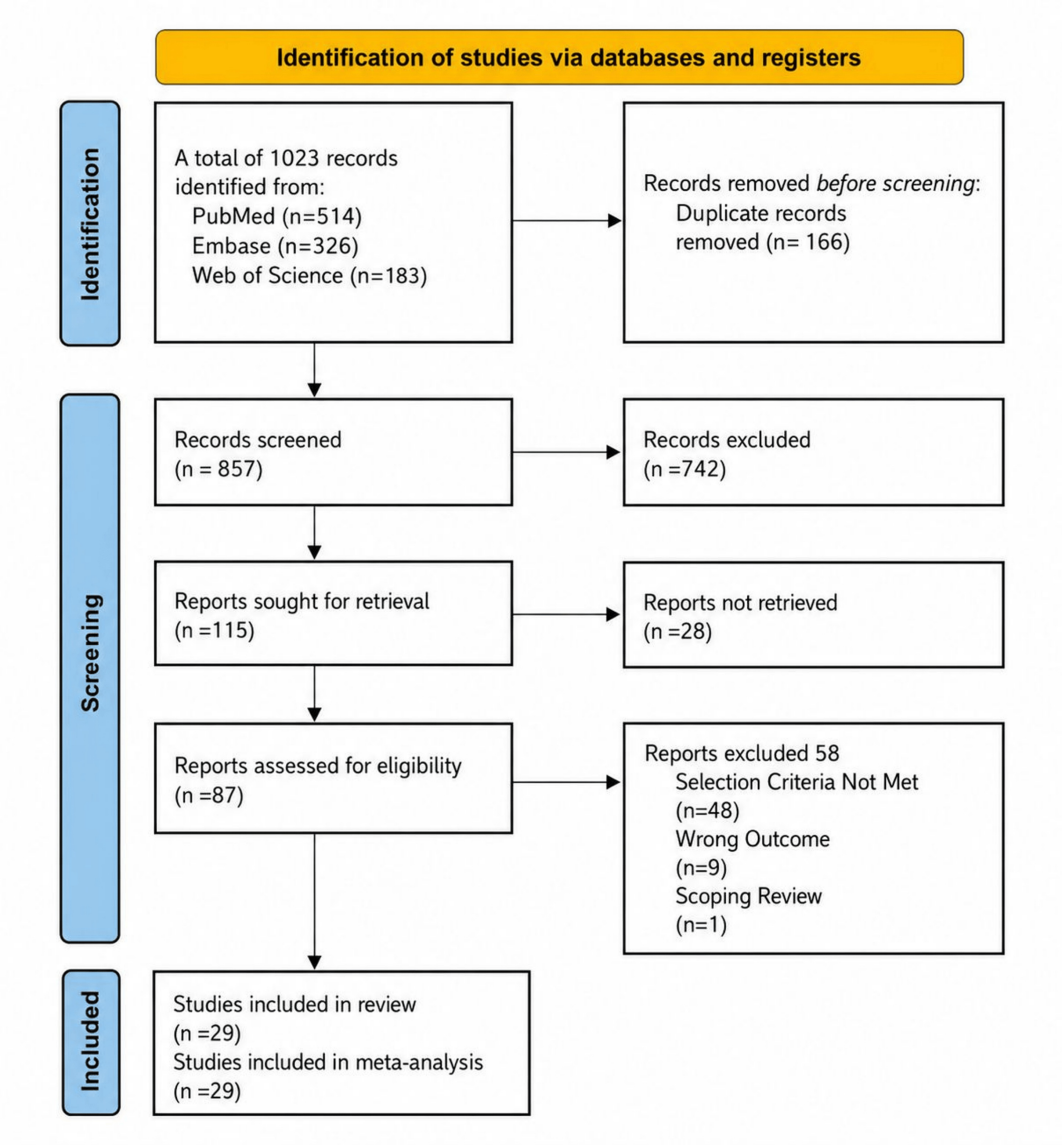
PRISMA flowchart depicting study selection PRISMA: Preferred Reporting Items for Systematic Reviews and Meta-Analyses

The statistical analysis utilized the “meta” package in RStudio and jamovi (Version 2.3), with statistical significance set at a p-value < 0.05. We used a random-effects meta-analysis model with inverse-variance weighting to account for between-study differences and estimate the overall prevalence, type-specific prevalence (oligomenorrhea, menorrhagia, polymenorrhea, hypomenorrhea, and metrorrhagia), and a secondary analysis of dysmenorrhea. We used I² statistics and Cochran's Q test to assess heterogeneity. An I² value of 75% or higher indicated substantial heterogeneity. To explore possible sources of variation, subgroup analyses were performed according to geographic location (North, South, East, and West India) and sample size (≤ 500 participants vs. ≥ 500 participants). Funnel plots and Egger's regression test were used to assess publication bias. A p-value of less than 0.05 indicated statistically significant publication bias (Figure 1).

Results

There were 1,023 records found through databases and registrations. After removing 166 duplicates, 857 records remained for screening. After the first screening, 742 records were excluded, and 115 full-text reports were requested for retrieval. Twenty-eight reports could not be retrieved; thus, 87 records remained for eligibility assessment (Figure 1). After evaluation, 58 papers were excluded, mostly because they did not meet the selection criteria (n = 48), reported improper findings (n = 9), or were scoping reviews (n = 1). In the end, 29 studies satisfied the inclusion criteria and were included in the systematic review (Table 3).

**Table 3:** Characteristics of included studies

Sr. No.	Study	State/union territory	Type of study/design	Sample size	Age group (years)	Abnormal uterine bleeding in participants
1.	Rani et al., 2016 [12]	Chandigarh	Cross-sectional	300	10-19	Dysmenorrhea: 184, oligomenorrhea, 94; polymenorrhea: 19
2.	Rathod et al., 2016 [13]	Maharashtra	Prospective and analytic study	655	11-19	Dysmenorrhea: 213, oligomenorrhea: 192, menorrhagia: 97
3.	Pegu et al., 2018 [14]	Andaman and Nicobar Islands	Descriptive analysis	824	10-19	Dysmenorrhea: 99, oligomenorrhea: 92, polymenorrhea: 75, menorrhagia: 138
4.	Dambhare et al., 2012 [15]	Maharashtra	Cross-sectional	561	10-19	Dysmenorrhea: 315, oligomenorrhea: 124, polymenorrhea: 47, hypermenorrhea: 7, hypomenorrhea: 9
5.	Tarannum et al., 2021 [16]	Uttar Pradesh	Cross-sectional	422	10-19	Oligomenorrhea: 119, polymenorrhea: 63, hypermenorrhea: 109, hypomenorrhea: 16, menorrhagia: 172, metrorrhagia: 185
6.	Wadgave et al., 2014 [17]	Maharashtra	Cross-sectional	400	13–19	Dysmenorrhea: 180, oligomenorrhea: 61, polymenorrhea: 33, menorrhagia: 55
7.	Sharma et al., 2015 [18]	New Delhi	Cohort	280	10-19	Dysmenorrhea: 20, oligomenorrhea: 30, menorrhagia: 82, metrorrhagia: 55
8.	Shringarpure et al., 2023 [19]	Gujarat	Cross-sectional	308	10-19	Dysmenorrhea: 118, oligomenorrhea: 43, menorrhagia: 26
9.	Verma and Baniya, 2022 [20]	Rajasthan	Cross-sectional	492	13-19	Dysmenorrhea: 318
10.	Madaan et al., 2014 [21]	Delhi	Cross-sectional	316	11-19	Dysmenorrhea: 118
11.	Singh et al., 2019 [22]	Delhi	Cross-sectional	210	10-19	Dysmenorrhea: 138, oligomenorrhea: 11, polymenorrhea: 11, hypermenorrhea: 53, hypomenorrhea: 9, menorrhagia: 53
12.	Agarwal et al., 2024 [23]	Bihar	Cross-sectional	2000	10-19	Dysmenorrhea: 315, oligomenorrhea: 215, polymenorrhea: 28, hypermenorrhea: 53, hypomenorrhea: 43, menorrhagia: 304, metrorrhagia: 184
13.	Singh et al., 2016 [24]	Uttar Pradesh	Cross-sectional	260	Age not mentioned, but includes adolescent girls	Dysmenorrhea: 123
14.	Sharma et al., 2008 [25]	Delhi	Cross-sectional	198	13-19 years	Dysmenorrhea: 133, hypomenorrhea: 9, menorrhagia: 27, hypermenorrhea: 30, oligomenorrhea: 63
15.	Mathiyalagen et al., 2017 [26]	Puducherry	Cross-sectional	242	12-18	Dysmenorrhea-199 Hypomenorrhea-5 Menorrhagia-55 Hypermenorrhea-51
16.	Mukherjee et al., 2014 [27]	West Bengal	Cross-sectional	159	15-19	Dysmenorrhea: 90, oligomenorrhea: 36, hypermenorrhea: 50
17.	Negi et al., 2018 [28]	Uttarakhand	Cross-sectional	470	13-19	Dysmenorrhea: 295, menorrhagia: 45, oligomenorrhea: 135
18.	Yaliwal et al., 2020 [29]	Karnataka	Cross-sectional	1016	10-19	Dysmenorrhea: 630, hypomenorrhea: 118, hypermenorrhea: 91, oligomenorrhea: 221, menorrhagia: 124
19.	Nidhi et al., 2011 [30]	Andhra Pradesh	Cohort	460	15-18	Oligomenorrhea: 72
20.	Ravi et al., 2017 [31]	Tamil Nadu	Cross-sectional	350	10-19	Dysmenorrhea: 254, menorrhagia: 160
21.	Juyal et al., 2023 [32]	Uttarakhand	Cross-sectional	453	15-16	Dysmenorrhea: 294, oligomenorrhea: 16, hypomenorrhea: 48
22.	Vani et al., 2013 [33]	Puducherry	Cross-sectional	853	13-19	Dysmenorrhea: 621, oligomenorrhea: 149
23.	Sanyal and Ray, 2008 [34]	West Bengal	Cross-sectional	220	14-18	Dysmenorrhea: 166, oligomenorrhea: 209, hypermenorrhea: 84
24.	Omidvar et al., 2015 [35]	Karnataka	Cross-sectional	536	10-19	Dysmenorrhea: 356, hypermenorrhea: 64, oligomenorrhea: 53, polymenorrhea: 79, menorrhagia: 159
25.	Ray et al., 2010 [36]	West Bengal	Cross-sectional	715	10-19	Dysmenorrhea: 218, oligomenorrhea: 639, menorrhagia: 131
26.	Thakre et al., 2012 [37]	Maharashtra	Cross-sectional	387	12-17	Dysmenorrhea: 236, hypomenorrhea: 4, menorrhagia: 35, oligomenorrhea: 58
27.	Sharanya 2014 [38]	Tamil Nadu	Cross-sectional	130	13-19	Dysmenorrhea: 79, polymenorrhea: 27, menorrhagia: 41
28.	Parikh and Nagar, 2022 [39]	Gujarat	Cross-sectional	127	10-19	Dysmenorrhea: 82, oligomenorrhea: 25, hypomenorrhea: 2, hypermenorrhea: 6, menorrhagia: 14
29.	Khan et al., 2024 [40]	Uttar Pradesh	Cross-sectional	640	10-19	Dysmenorrhea: 472, oligomenorrhea: 71, polymenorrhea; 65, hypomenorrhea: 179

The quality assessment of the included 30 studies was conducted using the Joanna Briggs Institute (JBI) Checklist for observational studies. Each study was scored across eight domains: (a) clear inclusion criteria, (b) detailed description of subjects and settings, (c) valid and reliable measurement of exposure, (d) use of standard diagnostic criteria for measuring exposure, (e) identification of confounders, (f) strategies to address confounding, (g) valid and reliable outcome measurement, and (h) appropriate statistical analysis. Items were scored as 1 (yes) or 0 (no), with total scores ranging from 3 to 7. Risk-of-bias assessments were performed independently by three authors, and the help of a fourth author was sought to resolve any conflicts that arose (Table 4).

**Table 4:** Quality assessment using JBI checklist JBI: Joanna Briggs Institute

Sr. No.	Study	1. Were the criteria for inclusion in the sample clearly defined?	2. Were the study subjects and the setting described in detail?	3. Was the exposure measured in a valid and reliable way?	4. Were objective, standard criteria used for measurement of the condition?	5. Were confounding factors identified?	6. Were strategies to deal with confounding factors stated?	7. Were the outcomes measured in a valid and reliable way?	8. Was appropriate statistical analysis used?	Total score
1.	Rani et al., 2016 [12]	1	1	1	1	0	0	1	1	6
2.	Rathod et al., 2016 [13]	1	1	1	1	0	0	1	1	6
3.	Pegu et al., 2018 [14]	1	1	0	0	0	0	0	1	3
4.	Dambhare et al., 2012 [15]	1	1	1	0	0	0	1	1	5
5.	Tarannum et al., 2021 [16]	1	1	1	0	1	1	1	1	7
6.	Wadgave et al., 2014 [17]	1	1	1	1	0	0	1	1	6
7.	Sharma et al., 2015 [18]	1	1	1	1	0	0	1	1	6
8.	Shringarpure et al., 2023 [19]	1	1	0	1	0	0	0	1	4
9.	Verma and Baniya, 2022 [20]	1	1	1	1	0	0	1	1	6
10.	Madaan et al., 2014 [21]	1	1	0	0	1	0	0	1	3
11.	Singh et al., 2019 [22]	1	1	1	0	1	0	0	1	5
12.	Agarwal et al., 2024 [23]	1	1	1	1	1	0	1	1	7
13.	Singh et al., 2016 [24]	1	1	1	1	0	0	1	1	6
14.	Sharma et al., 2008 [25]	1	1	1	0	0	0	1	1	5
15.	Mathiyalagen et al., 2017 [26]	1	1	1	0	0	0	1	1	5
16.	Mukherjee et al., 2014 [27]	1	1	1	0	1	1	0	1	6
17.	Negi et al., 2018 [28]	1	1	0	0	1	0	0	1	4
18.	Yaliwal et al., 2020 [29]	1	1	0	0	1	0	1	1	5
19.	Nidhi et al., 2011 [30]	1	1	1	1	0	0	1	1	6
20.	Ravi et al., 2017 [31]	1	1	1	1	0	0	1	1	6
21.	Juyal et al., 2023 [32]	1	1	1	1	0	0	1	1	6
22.	Vani et al., 2013 [33]	1	1	0	1	1	0	0	1	5
23.	Sanyal and Ray, 2008 [34]	1	1	1	0	1	1	0	1	6
24.	Omidvar et al., 2015 [35]	1	1	1	0	0	0	0	1	4
25.	Ray et al., 2010 [36]	1	1	0	0	1	1	0	1	5
26.	Thakre et al., 2012 [37]	1	1	0	0	1	1	0	1	5
27.	Sharanya, 2014 [38]	1	1	1	0	0	0	0	1	4
28.	Parikh and Nagar, 2022 [39]	1	1	0	0	0	0	0	1	3
29.	Khan et al., 2024 [40]	1	1	0	0	0	0	0	1	3

*Pooled Prevalence Estimates of Abnormal Uterine Bleeding (AUB) Among Adolescents in India* 

**Figure 2:**
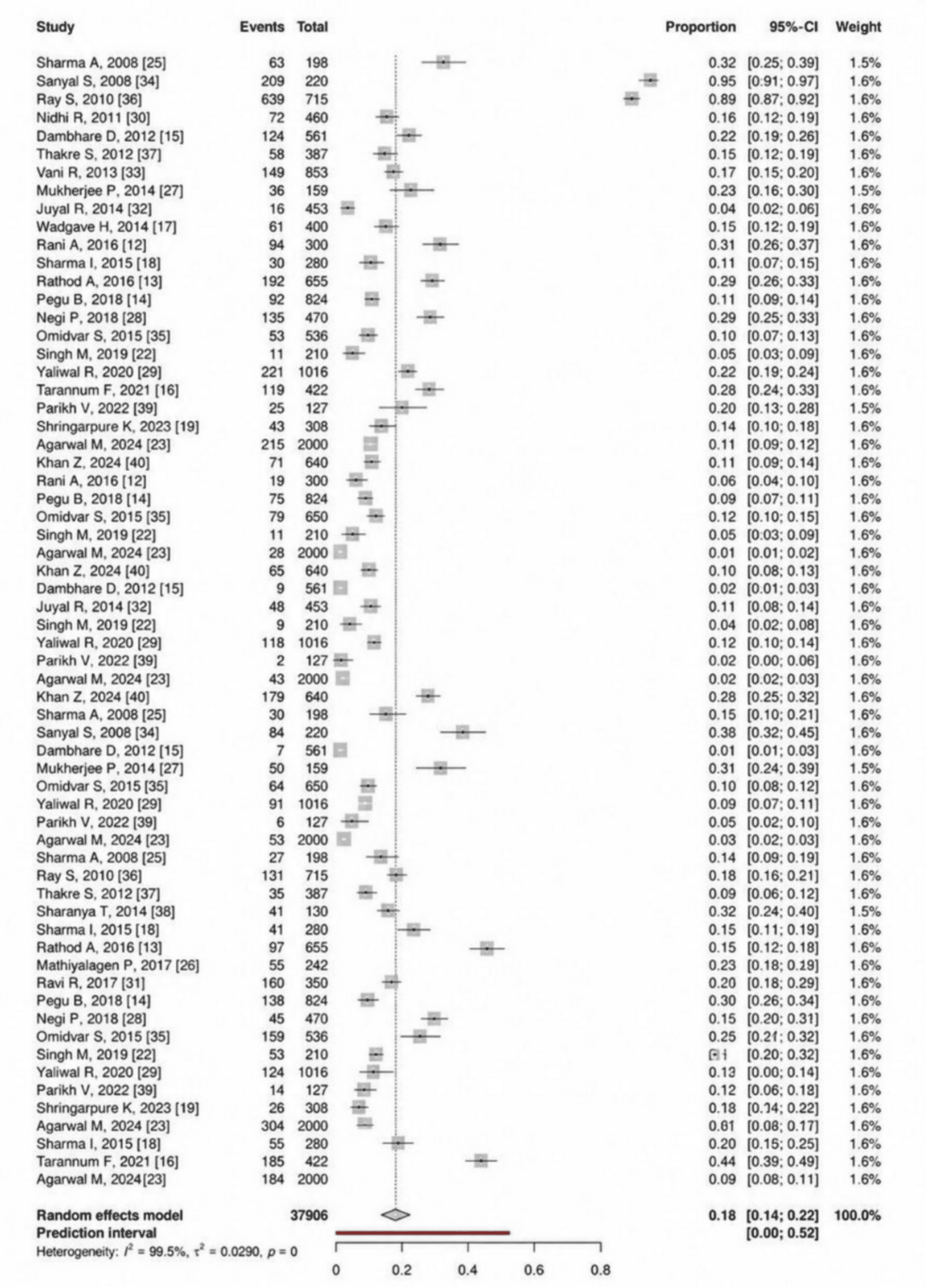
Forest plot showing the pooled prevalence of abnormal uterine bleeding ^#^Many studies appeared multiple times (if multiple abnormal uterine bleeding subtypes are reported in the same study)

A random‑effects meta‑analysis of 63 prevalence estimates derived from 29 studies yielded a pooled prevalence of AUB of 18.0% (95% CI: 14.0%-22.0%) in young girls aged 10-19 years. The overall estimate was statistically significant (Z = 8.47, p < 0.001), indicating a substantial burden of AUB across the included populations. Significant heterogeneity was observed among the studies (I² = 99.66%, Q = 12102.848, df = 62, p < 0.001). The estimated between‑studies variance (Tau² = 0.0286, SE = 0.0052; Tau = 0.169) further confirms substantial variability, which likely arises from differences in diagnostic criteria, geographic regions, and sample sizes. Consequently, the pooled prevalence should be interpreted with caution despite its statistical significance (Figure 2).

Publication bias assessment

**Figure 3:**
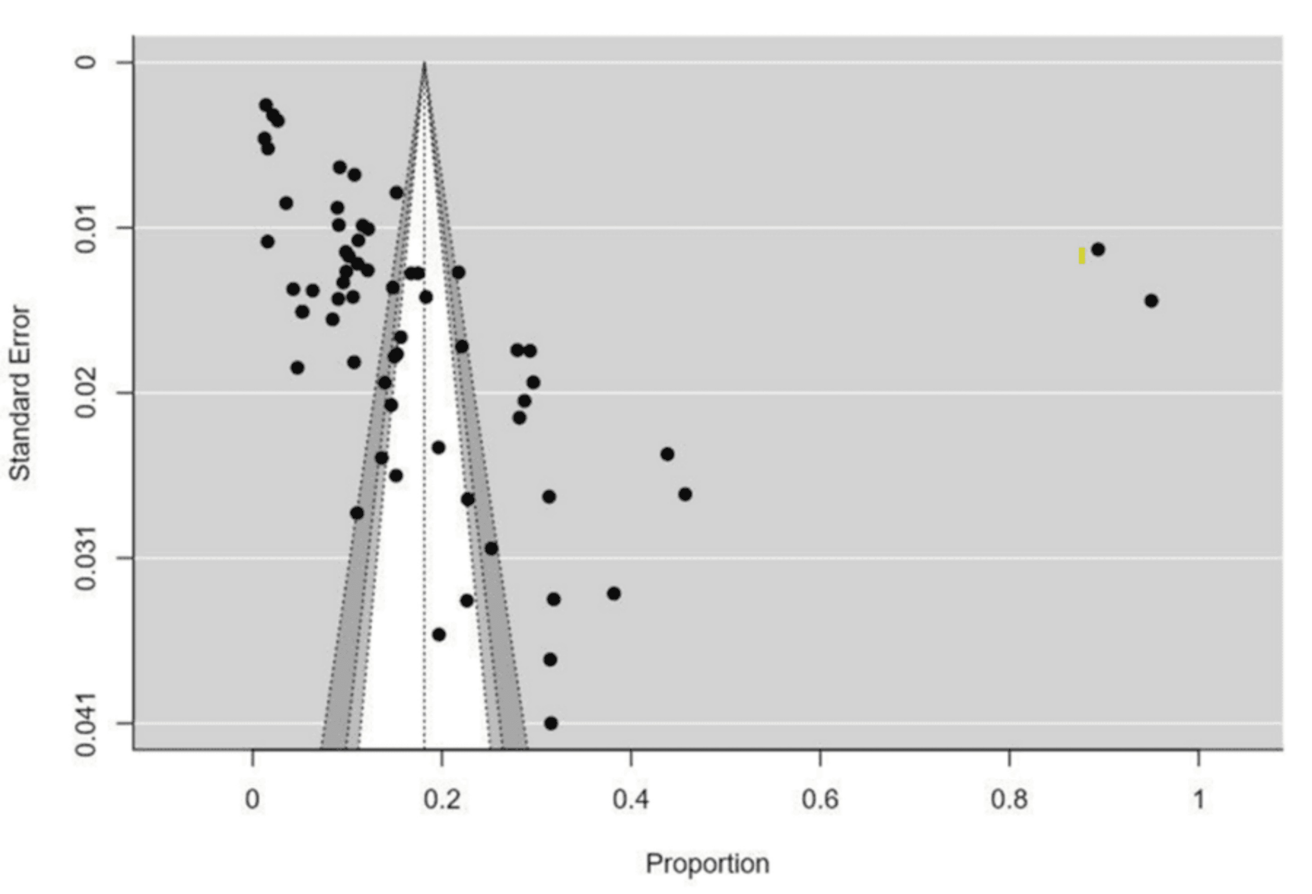
Funnel plot for the assessment of publication bias in the pooled prevalence of abnormal uterine bleeding

Evaluation of publication bias revealed funnel plot asymmetry. The rank correlation test (Kendall’s τ = 0.314, p < 0.001) and the regression test (Z = 3.172, p = 0.002) both suggested the presence of small-study effects. However, the fail-safe N (Rosenthal method) was 179,132 (p < 0.001), indicating that an extremely large number of null or unpublished studies would be required to overturn the observed pooled estimate. Thus, despite the asymmetry, the overall prevalence estimate appears robust to potential publication bias. The forest plot (Figure 2) illustrates the distribution of individual study estimates, most of which fell within the calculated confidence interval. The funnel plot (Figure 3) visually confirms the asymmetry identified by the statistical tests.

Pooled Prevalence Estimates for Several Types of Abnormal Uterine Bleeding (AUB) Among Adolescent Girls in India

**Figure 4:**
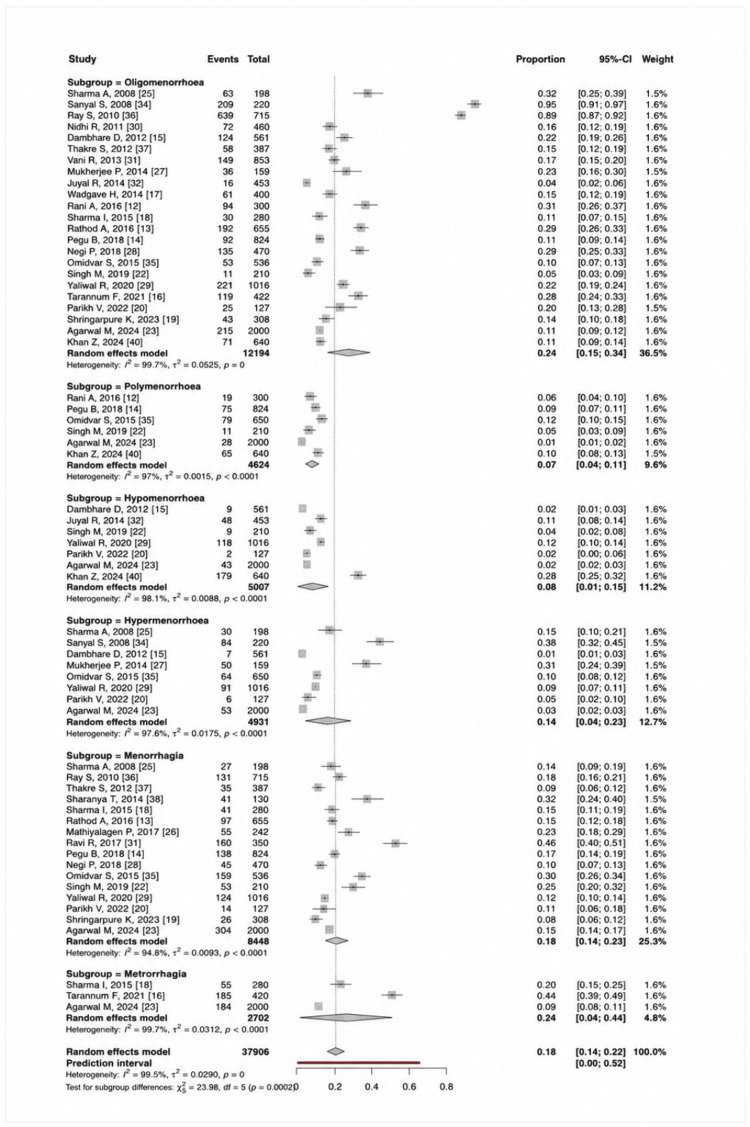
Subgroup analysis and pooled prevalence for various types of abnormal uterine bleeding

Our subgroup analysis of AUB subtypes revealed significant variation in the pooled prevalence among Indian young girls across different states. Oligomenorrhea showed the highest pooled prevalence at 24% (95% CI: 15-34%) (Figure 4), with significant heterogeneity (I² = 99.7%, τ² = 0.0525, p = 0). The highest prevalence was reported by Sanyal et al. (95%) [34], and the lowest by Juyal et al. (4%) [32]. Polymenorrhea had a pooled prevalence of 7% (95% CI: 4-11%), with high heterogeneity (I² = 97%, τ² = 0.0015, p < 0.0001). Prevalence ranged from 1% (Agarwal et al. [23]) to 12% (Omidvar et al. [35]). Hypomenorrhea had a pooled prevalence of 8% (95% CI: 1-15%), with considerable heterogeneity (I² = 98.1%, τ² = 0.0088, p < 0.0001). The lowest prevalence was 2%, reported by Dambhare et al. [15] and Parikh et al. [41], and the highest was 28% (Agarwal et al. [23]). Hypermenorrhea showed a pooled prevalence of 14% (95% CI: 4-23%), with substantial heterogeneity (I² = 97.6%, τ² = 0.0175, p < 0.0001). The prevalence ranged from 1% (Dambhare et al. [15]) to 38% (Sanyal et al. [34]). Menorrhagia had a pooled prevalence of 18% (95% CI: 14-23%), with significant heterogeneity (I² = 94.8%, τ² = 0.0093, p < 0.0001). Prevalence varied from 8% (Shringarpure et al. [19]) to 46% (Ravi R. et al. [31]). All AUB subtypes demonstrated very high heterogeneity (I² ≥ 94.8%), indicating substantial variation across studies.

Subgroup Analysis by Geographical Region

**Figure 5:**
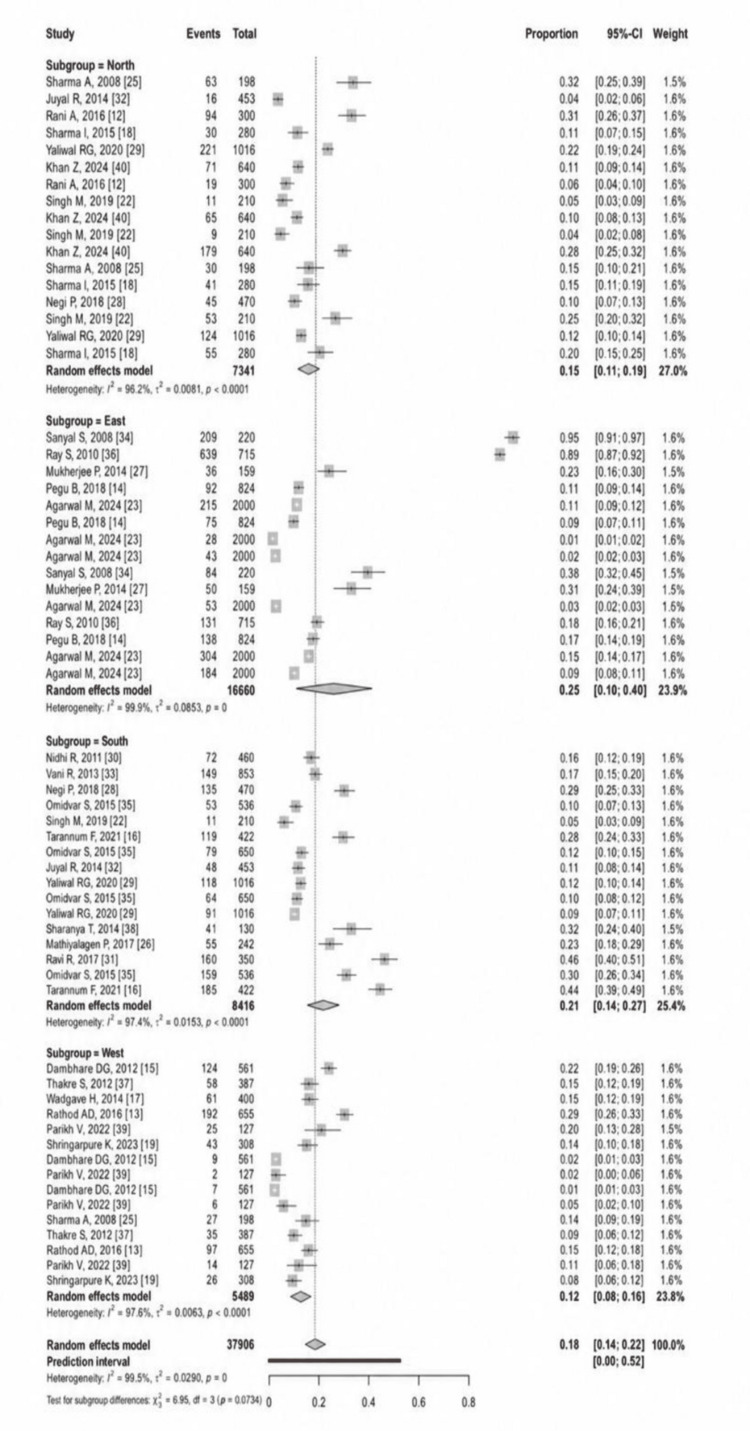
Subgroup analysis by geographical region

Subgroup analysis by geographical region revealed no statistically significant difference in the pooled prevalence of AUB among Indian young women across different states (Figure 5). The analysis showed substantial heterogeneity (I² = 99.5%, p < 0.001). For the North region, individual study proportions ranged from 4% to 32%, with the lowest prevalence (4%) reported by Juyal et al. [32] and the highest (32%) by Sharma et al. [25]. Heterogeneity in this region was substantial (I² = 96.2%, τ² = 0.0081, p < 0.0001). In the East region, study proportions ranged from 1% to 95%, with an extreme outlier. Sanyal et al. [34] reported a 95% prevalence of oligomenorrhea, whereas the lowest prevalence (1%) was reported by Agarwal et al. [23].

Heterogeneity was extremely high (I² = 99.9%, τ² = 0.0853, p < 0.001). For the South region, proportions varied from 5% to 46%, with the highest (46%) reported by Ravi R. et al. [31] and the lowest (5%) by Singh et al. [22]. Heterogeneity was also significant (I² = 97.4%, τ² = 0.0153, p < 0.0001). In the West region, study proportions ranged from 1% to 29%, with the highest (29%) reported by Rathod et al. [13] and the lowest (1%) by Dambhare et al. [15]. Heterogeneity was also high (I² = 97.6%, τ² = 0.0063, p < 0.0001). The considerable regional differences, particularly the 95% prevalence reported in the East, suggest that geographical factors (e.g., healthcare access and operational definitions), methodological factors, and diagnostic factors contribute to the observed variance.

Subgroup Analysis by Sample Size

**Figure 6:**
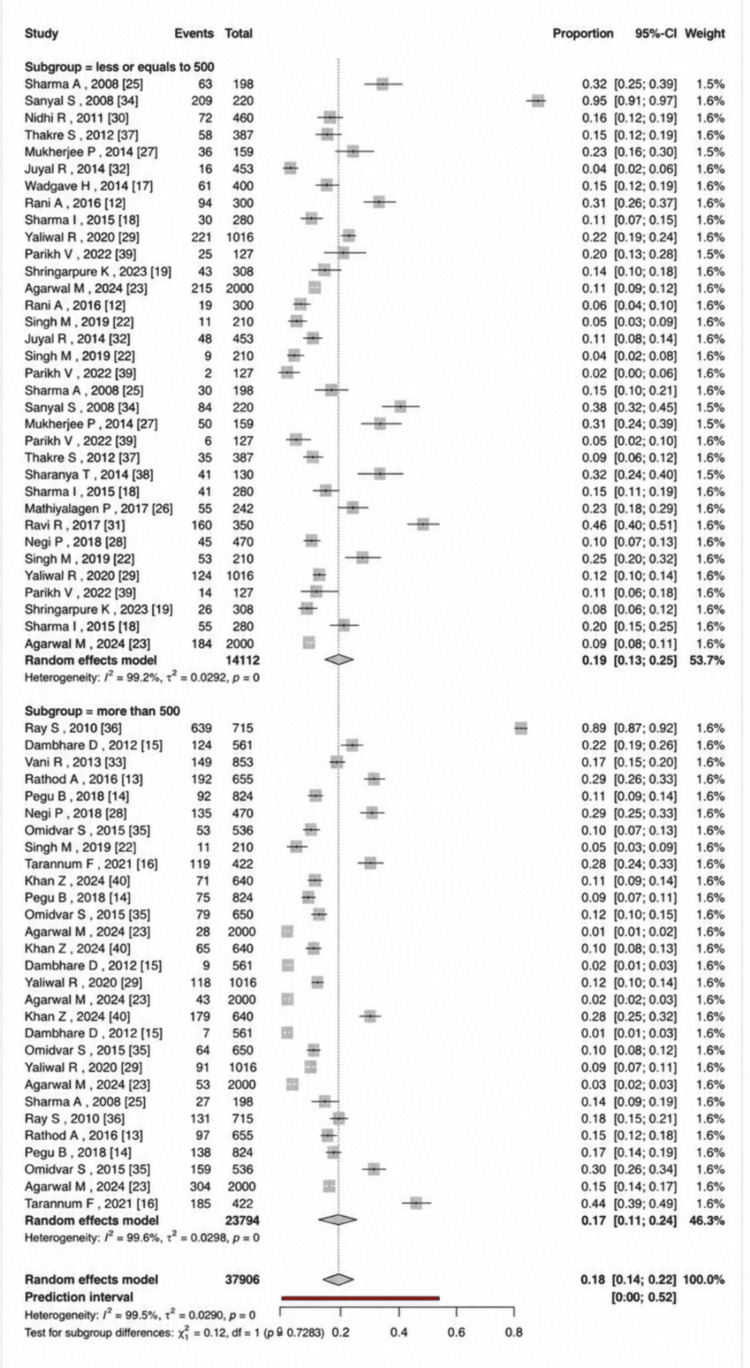
Subgroup analysis by sample size

The subgroup analysis compared studies on the basis of sample size. In this sample sizes ≤ 500 versus those studies with sample size > 500 yielded no statistically significant difference (χ² = 0.12, df = 10, p = 0.728), or we can say, sample size does not significantly influence prevalence estimates (Figure 6) Both subgroups demonstrated extremely high heterogeneity (sample size ≤ 500: I² = 99.2%; sample size > 500: I² = 99.6%; overall: I² = 99.5%), indicating substantial variability across studies. The random effects model for the entire set also showed high heterogeneity (τ² = 0.0290, p < 0.001), explaining amount of variability between studies is substantial. Overall, the subgroup difference was not significant, suggesting that sample size category (≤ 500 vs. >500) did not modify the pooled proportion for AUB (Figure 6).

A sensitivity analysis excluding studies with a JBI quality score of ≤ 3 showed a pooled prevalence of 20% (95% CI: 15%-25%), with no substantial change compared to the primary analysis, indicating that the overall pooled estimate was robust and not driven by lower-quality studies.

Burden Due to the Most Common Menstrual Morbidity Among Adolescent Girls in India: Dysmenorrhea

**Figure 7:**
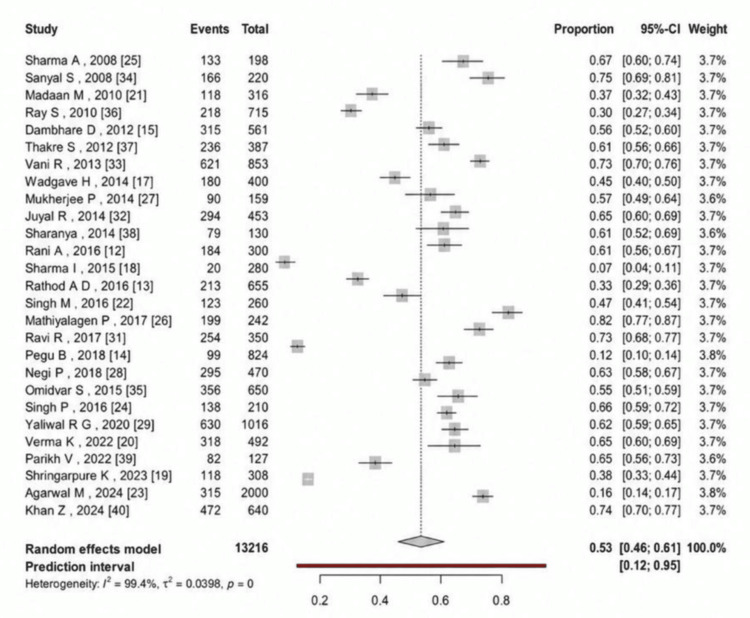
Forest plot showing the pooled prevalence of dysmenorrhea among adolescent girls in India

The pooled prevalence of dysmenorrhea in adolescent females was 53% (95% CI: 46-61%, I² = 99.4%), with individual study estimates ranging from 12% to 82% (Figure 7).

Discussion

This systematic review and meta-analysis provides the most comprehensive synthesis of the currently available evidence on AUB among adolescent girls in India. However, because of substantial heterogeneity, regional variation, and methodological differences among the included studies, the pooled prevalence should be interpreted as an approximate summary of the existing evidence rather than a stable national estimate. The pooled prevalence of AUB in this study was 18.0% (95% CI: 14%-22%), underscoring AUB as a significant public health issue in India. In comparison, a global study conducted in Egypt among rural adolescent girls reported an AUB (FIGO System 1) prevalence of 64.8% [41], while another large study conducted in Singapore among adolescents reported a menstrual irregularity prevalence of 23.1%, which was close to the findings of our study [42].

Evaluating AUB has always posed challenges, primarily due to its complex nature, which encompasses a wide range of symptoms and subtypes. The lack of standardized assessment tools and reliance on self-reported data from participants further complicate the evaluation process. Future large, nationally representative studies using standardized FIGO-based definitions are needed to generate more precise national prevalence estimates. 

Our subgroup analysis of AUB subtypes sheds light on the epidemiological patterns within India, providing valuable insights for targeted screening and effective referral management. The three most prevalent AUB subtypes identified in this systematic review were oligomenorrhea (24%), metrorrhagia (24%), and menorrhagia (18%). The cultural silence and stigma surrounding menstrual health in India and other parts of the world contribute to the underreporting of these conditions, despite their association with various underlying health issues [43]. Addressing these cultural barriers is essential for improving awareness, encouraging open discussions, and facilitating better management of AUB among adolescents. By prioritizing education and support, we can enhance the detection and treatment of AUB, ultimately promoting better reproductive health outcomes for young women.

In our study, we found the pooled estimate of oligomenorrhea to be 24%. In contrast, a systematic review from Iran reported a pooled prevalence of only 13.1%, which was significantly lower than our findings [44]. Additionally, a large-scale study focusing on an Italian adolescent female population found the prevalence of oligomenorrhea to be just 3.4% [45]. Interestingly, another study examining young female athletes revealed a high prevalence of 24%, which closely aligned with our results [46]. Oligomenorrhea, characterized by infrequent menstrual periods, is a significant gynecological issue that can cause considerable anxiety among students and those around them. Beyond the emotional impact, oligomenorrhea is associated with various health risks, including infertility, cardiovascular complications, and metabolic disturbances. Conditions such as obesity, hyperandrogenism, and metabolic syndrome are linked to this menstrual irregularity, highlighting the importance of addressing oligomenorrhea not only as a reproductive health concern but also as a broader public health issue [47,48].

Our study reported pooled prevalence estimates of 18% for menorrhagia and 14% for hypermenorrhea. In contrast, a systematic review and meta-analysis conducted in Iran found pooled prevalence estimates of 13.1% for menorrhagia and 12.4% for hypermenorrhea [44]. Additionally, a study conducted in Canada among adult and adolescent women revealed a pooled estimate of 30% for heavy menstrual bleeding (HMB) [49]. It is important to note that HMB is defined as heavy bleeding exceeding 80 mL and encompasses both menorrhagia and hypermenorrhea. Interestingly, a cohort study from the Netherlands reported a much lower incidence rate of HMB, highlighting regional variations in prevalence [50]. Furthermore, another systematic review conducted across ten Asian countries indicated a significantly higher pooled estimate of 48.6% for HMB [51].

These findings collectively suggest a notable discrepancy in the prevalence of HMB between developing and developed nations. The higher prevalence rates observed in developing countries may reflect limited health-seeking behavior, restricted access to healthcare resources, and the absence of standardized screening tools for menstrual disorders. Cultural stigma and insufficient education surrounding menstrual health further hinder the proper management and referral of these conditions. By addressing these barriers, we can improve the identification and treatment of menstrual disorders, ultimately enhancing the quality of life for affected individuals. Overall, the comparison of results across multiple studies highlights the need for increased awareness, education, and the implementation of standardized screening practices in developing nations like India. Other systematic reviews and meta-analyses have identified the three most common etiologies for HMB as von Willebrand disease, platelet function defects, and thyroid disease, further highlighting the severity of this problem [49].

Various clinical abnormal bleeding patterns that constitute AUB (FIGO System 1) include menorrhagia, polymenorrhea, oligomenorrhea, irregular menstruation, and others. These conditions are a major source of absenteeism and poor academic performance among young women. Rafique et al. observed that menstrual problems such as menorrhagia, irregular uterine bleeding, and polymenorrhea accounted for over 12% of gynecology referrals and were associated with a high rate of surgical intervention among young females [52]. Among menstrual disorders, dysmenorrhea is a major health concern for women worldwide. Dysmenorrhea is a common condition among adolescent girls, affecting their quality of life, work productivity, and healthcare utilization throughout the reproductive years [53]. A recent systematic review conducted among Indian women reported the prevalence of dysmenorrhea ranging from 46% to 76%, which was consistent with our study findings. The pooled prevalence of dysmenorrhea was 53% (95% CI: 46-61%, I² = 99.4%) [54].

In contrast, a higher pooled prevalence estimate of dysmenorrhea was observed among female students in Ethiopia, with a prevalence of 71.69% (95% CI: 66.82-76.56%), with significant heterogeneity (I² = 94.2%, p < 0.001) [53]. This difference can be attributed to several sociodemographic, cultural, and methodological factors. One of the primary reasons could be differences in the study populations. Variations in awareness levels and health-seeking behavior, including access to menstrual health education, might have also influenced these findings. Another systematic review and meta-analysis conducted in Iran estimated a combined prevalence of primary dysmenorrhea of 73.27% (95% CI: 65.12%-81.42%), which was also higher than our findings [44]. Their analysis showed considerable heterogeneity among studies (Q = 4097.93, df = 25, p < 0.001, and I² = 99.4%). An individual study conducted in the Ghaziabad district among women aged 15-45 years residing in an urban area found the prevalence of primary dysmenorrhea to be 67.8%, which was consistent with our study results [55].

Polymenorrhea is a menstrual irregularity characterized by menstrual cycles occurring more frequently than normal, with an interval of less than 21 days [44]. A recent systematic review conducted among Indian women estimated the prevalence of polymenorrhea to range from 3% to 22.2%, which aligned with our findings, where the pooled prevalence was estimated at 7% (95% CI: 4-11%, range: 1-12%) [54]. A study conducted in Kerala estimated the prevalence at 3.1% [56], while 9.94% of women in Iran reported experiencing frequent periods [44]. Similarly, a lower prevalence of polymenorrhea was observed in most previous studies [54].

Hypomenorrhea is a disorder characterized by light uterine bleeding, defined by a reduced menstrual flow in terms of duration, number of bleeding days (< 2 days), or both [57]. Our study estimated a pooled prevalence of 8% (95% CI: 0.01-0.15%), with high heterogeneity (I² = 98.1%, τ² = 0.0088, p < 0.0001) among Indian adolescent girls. The findings of Das et al., which reported a prevalence of 5.1% in an individual study, were within our estimated range [54]. The prevalence of hypomenorrhea in Iran, as reported in a systematic review, was 5.25% (95% CI: 3.20%-7.30%), with estimates ranging from 0.9% to 12.90% across the studies included in the analysis [44]. The literature indicates that hypomenorrhea is often underrecognized, particularly among adolescents, where its identification is not routinely practiced despite its potential implications for menstrual health [45].

A systematic review and meta-analysis conducted in Iran reported a pooled prevalence of metrorrhagia of 6.04% (95% CI: 1.99-10.08%), which was considerably lower than the 24% estimated in our study [44]. In contrast, a cross-sectional study conducted among secondary school girls in Nigeria reported a higher prevalence of 18.6%, which was closer to our findings and may reflect similarities in adolescent-focused populations and study settings [58]. Differences in the study populations could account for the variation in findings, as the Iranian meta-analysis included a wider age range than our study, which focused primarily on adolescents who are more prone to anovulatory cycles.

There are only a few Indian meta-analyses that provide pooled prevalence estimates for metrorrhagia among adolescents, while larger reviews and individual studies have demonstrated significant heterogeneity. In a two-decade assessment of menstrual disorders in India, prevalence estimates ranged from 3% to 87%, indicating variations in classifications, study settings, and demographic characteristics [54]. A study conducted in Puducherry found metrorrhagia in 1.1% of adolescents with menstrual disorders [36]. These results show that our study’s pooled estimate of 24% (95% CI: 4-44%), though higher than clinical estimates, is compatible with community-level data, especially when broader menstrual abnormalities, self-reported symptoms, and adolescent-specific variables are considered.

Limitations

There are certain limitations associated with this review. Firstly, the high statistical heterogeneity (I² > 90%) observed across all analyses limits the reliability and generalizability of the pooled estimates of AUB and its subtypes among Indian adolescent girls. The second limitation is the lack of standardized methods, assessment tools, or diagnostic criteria for data collection, which may have directly influenced the pooled estimates. A third important limitation is that a substantial proportion of the included studies did not adjust for important confounding factors that may influence the prevalence and subtypes of abnormal uterine bleeding among adolescent girls. These factors include anthropometric parameters such as BMI, nutritional status, dietary patterns, physical activity levels, socioeconomic status, anemia or hemoglobin levels, and underlying medical conditions such as PCOS, thyroid disorders, and bleeding diatheses. This limitation restricted the ability of the study to perform meta-regression. The absence of multivariable adjustment in most studies may have influenced the observed prevalence estimates of AUB without accounting for these potential confounders. Additionally, biases attributed to language restrictions, searches limited to peer-reviewed journals, variations in study settings, and differences in data collection methods were not explored in this study, which may have limited the ability to fully capture the true burden of AUB among adolescent girls.

## Conclusions

This systematic review and meta-analysis provides the first comprehensive national synthesis of AUB and its clinical subtypes among adolescent girls in India. Approximately one in five adolescents experienced AUB, while dysmenorrhea affected more than half of the study population, highlighting the considerable burden of menstrual health problems in this age group. Oligomenorrhea and metrorrhagia emerged as the most prevalent AUB subtypes, followed by menorrhagia. Although substantial heterogeneity was observed across studies, likely reflecting regional, methodological, and diagnostic differences, the findings consistently indicate that menstrual disorders represent an important but underrecognized public health concern. Thus, this study supports the need for future studies to evaluate the effectiveness, accuracy, and feasibility of screening strategies for their broader implementation.

## Appendices

**Table 5:** PRISMA 2020 checklist PRISMA: Preferred Reporting Items for Systematic Reviews and Meta-Analyses

Section and topic	Item#	Checklist item	Location where item is reported
TITLE			
Title	1	Identify the report as a systematic review	Page 1
ABSTRACT			
Abstract	2	See the PRISMA 2020 for Abstracts checklist	Page 1
INTRODUCTION			
Rationale	3	Describe the rationale for the review in the context of existing knowledge	Introduction (pages 1-2)
Objectives	4	Provide an explicit statement of the objective(s) or question(s) the review addresses	(page 2)
METHODS			
Eligibility criteria	5	Specify the inclusion and exclusion criteria for the review and how studies were grouped for the syntheses	Eligibility criteria (page 3)
Informationsources	6	Specify all databases, registers, websites, organisations, reference lists and other sources searched or consulted to identify studies. Specify the date when each source was last searched or consulted	Data sources and search strategy (pages 2-4)
Search strategy	7	Present the full search strategies for all databases, registers and websites, including any filters and limits used	Table 2 (pages 3-4)
Selection process	8	Specify the methods used to decide whether a study met the inclusion criteria of the review, including how many reviewers screened each record and each report retrieved, whether they worked independently, and if applicable, details of automation tools used in the process	Data extraction using Rayyan software (page 5)
Data collection process	9	Specify the methods used to collect data from reports, including how many reviewers collected data from each report, whether they worked independently, any processes for obtaining or confirming data from study investigators, and if applicable, details of automation tools used in the process	Data extraction using Rayyan software (page 4)
Data items	10a	List and define all outcomes for which data were sought. Specify whether all results that were compatible with each outcome domain in each study were sought (e.g. for all measures, time points, analyses), and if not, the methods used to decide which results to collect	Data extraction using Rayyan software (page 4)
	10b	List and define all other variables for which data were sought (e.g. participant and intervention characteristics, funding sources). Describe any assumptions made about any missing or unclear information	Data extraction using Rayyan software, Table 1 (pages 2 and 4)
Study risk of bias assessment	11	Specify the methods used to assess risk of bias in the included studies, including details of the tool(s) used, how many reviewers assessed each study and whether they worked independently, and if applicable, details of automation tools used in the process	JBI checklist, Table 4
Effect measures	12	Specify for each outcome the effect measure(s) (e.g. risk ratio, mean difference) used in the synthesis or presentation of results	Data analysis and results (page 4) (Figures 2-7)
Synthesis methods	13a	Describe the processes used to decide which studies were eligible for each synthesis (e.g. tabulating the study intervention characteristics and comparing against the planned groups for each synthesis (item #5)).	Data extraction analysis (page 4)
	13b	Describe any methods required to prepare the data for presentation or synthesis, such as handling of missing summary statistics, or data conversions	Not reported
	13c	Describe any methods used to tabulate or visually display results of individual studies and syntheses	Data analysis (page 4)
	13d	Describe any methods used to synthesize results and provide a rationale for the choice(s). If meta-analysis was performed, describe the model(s), method(s) to identify the presence and extent of statistical heterogeneity, and software package(s) used	Data analysis (page 4)
	13e	Describe any methods used to explore possible causes of heterogeneity among study results (e.g. subgroup analysis, meta-regression)	Data analysis (page 4)
	13f	Describe any sensitivity analyses conducted to assess robustness of the synthesized results	Data analysis (page 4)
Reporting bias assessment	14	Describe any methods used to assess risk of bias due to missing results in asynthesis (arising from reporting biases)	Publication bias, funnel plot and Egger’s plot (pages 9-10, Figure 3)
Certainty assessment	15	Describe any methods used to assess certainty (or confidence) in the body of evidence for an outcome	Not reported (GRADE not performed)
